# Bringing Attomolar Detection to the Point‐of‐Care with Nanopatterned DNA Origami Nanoantennas

**DOI:** 10.1002/adma.202507407

**Published:** 2025-07-26

**Authors:** Renukka Yaadav, Kateryna Trofymchuk, Mihir Dass, Vivien Behrendt, Benedikt Hauer, Jan Schütz, Cindy Close, Michael Scheckenbach, Giovanni Ferrari, Leoni Mäurer, Sophia Sebina, Viktorija Glembockyte, Tim Liedl, Philip Tinnefeld

**Affiliations:** ^1^ Department of Chemistry and Center for NanoScience Ludwig‐Maximilians‐University Butenandtstraße 5−13 81377 Munich Germany; ^2^ Faculty of Physics and Center for NanoScience Ludwig‐Maximilians‐University 80539 Munich Germany; ^3^ Fraunhofer Institute for Physical Measurement Techniques IPM Georges‐Köhler‐Allee 301 79110 Freiburg Germany

**Keywords:** biosensing, DNA origami nanoantenna, fluorescence enhancement, point‐of‐care, single‐molecule detection

## Abstract

Creating increasingly sensitive and cost‐effective nucleic acid detection methods is critical for enhancing point‐of‐care (POC) applications. This requires highly specific capture of biomarkers and efficient transduction of capture events. However, the signal from biomarkers present at extremely low amounts often falls below the detection limit of typical fluorescence‐based methods, necessitating molecular amplification. Here, we present single‐molecule detection of a non‐amplified, 151‐nucleotide sequence specific to antibiotics‐resistant *Klebsiella pneumoniae* down to attomolar concentrations, using Trident NanoAntennas with Cleared HOtSpots (NACHOS). This NACHOS‐diagnostics assay leverages a compact microscope with a large field‐of‐view, including microfluidic flow to enhance capturing efficiency. Fluorescence enhancement is provided by NanoAntennas, arranged using a combination of nanosphere lithography and site‐specific DNA origami placement. This method can detect 200 ± 50 out of 600 molecules in a 100 µL sample volume within an hour. This represents a typical number of pathogens in clinical samples commonly detected by Polymerase Chain Reaction. We achieve similar sensitivity in untreated human plasma, enhancing the practical applicability of the system. This platform can be adapted to detect shorter nucleic acid fragments that are not compatible with traditional amplification‐based technologies. This provides a robust and scalable solution for sensitive nucleic acid detection in diverse clinical settings.

## Graphical abstract

Single DNA molecules are captured within the hotspots of DNA origami NanoAntennas arranged on a chip surface. Fluorescence is amplified, enabling direct detection on a point‐of‐care‐compatible imaging setup (visualized as bright signals against a dark background).

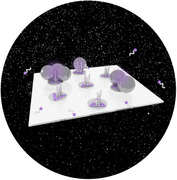

## 1 Introduction

Single‐molecule methods are gaining ground in bioanalytical applications like nucleic acid sequencing and sensitive analyte detection.^[^ ^1^, ^2^, ^3^, ^4^ ^]^ For Point‐of‐Care (POC) detection in low‐technology environments, however, single‐molecule approaches are still considered prohibitively expensive due to their reliance on sophisticated setups. For instance, single‐molecule detection using fluorescence often requires either molecular multiplication to detect the target's signal against background noise or expensive instrumentation to detect single fluorescent molecules directly.^[^ ^1^, ^5^, ^6^ ^]^ Advances in plasmonic fluorescence enhancement,^[^ ^7^, ^8^, ^9^ ^]^ utilizing DNA origami nanostructures,^[^ ^10^, ^11^, ^12^ ^]^ have facilitated signal amplification of fluorophores captured in the hotspot, improving the distinction of the real signal from background impurities and enabling single‐molecule detection using a portable, battery‐driven smartphone microscope.^[^ ^13^ ^]^ But their use for target detection at clinically relevant nucleic acid concentrations below the picomolar range^[^ ^14^ ^]^ has remained elusive.

Arguably, analytical methods cannot get more sensitive than detecting a single molecule, and concentration determination cannot become more direct than digitally counting all molecules in a sample.^[^ ^2^, ^15^, ^16^, ^17^ ^]^ Single‐molecule detection is thus swiftly moving toward the development of affordable, portable devices, making it accessible outside of specialized laboratories and into POC and field settings.^[^ ^13^, ^18^, ^19^, ^20^, ^21^, ^22^, ^23^ ^]^ The challenge here is not to detect the single molecule present in a (typically very small) detection volume, but to find all molecules in the patient sample. At 1 aM (10^−18^ mol L^−1^) concentration, 100 µL of blood serum contains ≈60 molecules that need to be detected. It is impractical to rely solely on Brownian motion to transport these molecules through the minuscule detection volumes of, e.g., a focused laser beam or a nanopore.^[^ ^1^ ^]^ To address this, most detection methods rely on a pre‐concentration step^[^ ^2^, ^24^, ^25^ ^]^ or utilize molecular amplification strategies.^[^ ^26^, ^27^, ^28^ ^]^ For nucleic acids, dPCR (digital polymerase chain reaction) has been a game changer. dPCR works by partitioning the sample into many reaction chambers or droplets and diluting until each chamber contains one or zero target molecules. The molecules are then amplified by thermal cycling, and the positive droplets are detected using fluorescence‐based methods, determining an absolute number of target nucleic acids.^[^ ^20^ ^]^ However, practical use of commercially available dPCR devices, especially in low‐resource settings, is still limited by complex workflow, the need for trained personnel, and advanced equipment.^[^ ^29^ ^]^

Building on addressable NanoAntennas with Cleared HOtSpots (NACHOS),^[^ ^13^ ^]^ we present NACHOS‐diagnostics, a POC‐compatible amplification‐free detection approach for nucleic acids that addresses these challenges. Specifically, we target a synthetic DNA sequence specific to carbapenem‐resistant *Klebsiella pneumoniae*, a bacterium which has been directly linked to 1.27 million deaths and contributing to 4.95 million deaths annually.^[^ ^30^ ^]^ The fluorescence enhancement by NanoAntennas not only facilitates single‐molecule detection with simple optics, but it also creates a contrast against an unavoidable background of single‐molecule impurities, thus minimizing false positive signals.^[^ ^13^ ^]^ NACHOS diagnostics utilizes a sandwich assay^[^ ^13^ ^]^ with a capturing sequence and a dye‐labeled imager strand to detect DNA target strands over a broad concentration range from attomolar to nanomolar.

## 2 Results

**Figure 1:**
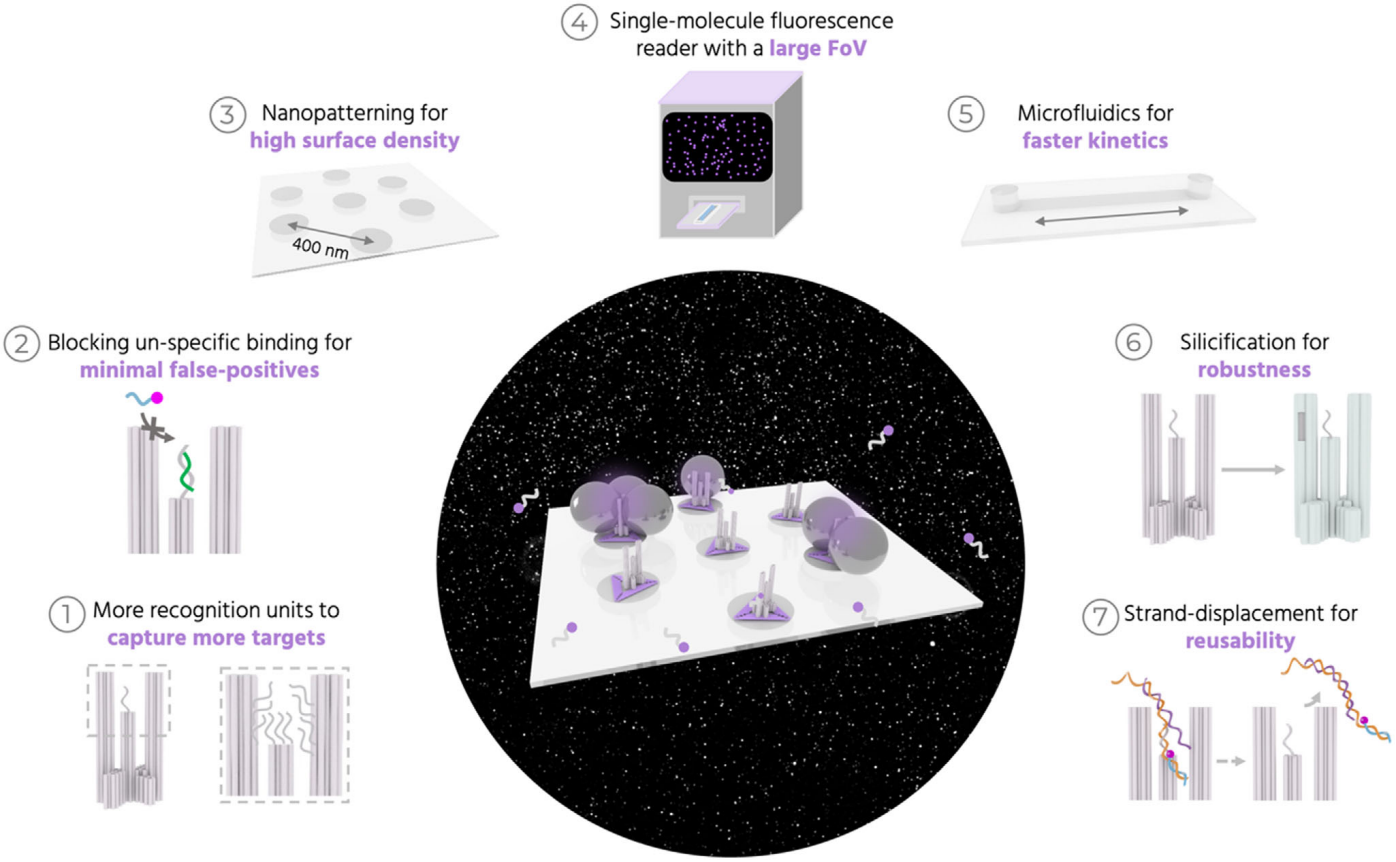
Overview of the different components for NACHOS diagnostics. Different components involved in our approach to develop fluorescence enhanced single‐molecule detection into a POC‐compatible method for detecting low target concentrations of nucleic acids. An abstract image in the center shows a purple glow upon capturing DNA in the NanoAntennas, with a snapshot of thousands of single molecules detected on our fluorescence reader as the background. Panels 1–7 show the different steps we employed to address the challenges in achieving high sensitivity.

At nucleic acid concentrations where only a few molecules are present in the sample volume, detection systems face significant challenges− background noise from impurities, sensitivity of the assay, and long response times.^[^ ^24^ ^]^ Employing NanoAntennas allows us to tackle the first challenge by physically amplifying the fluorescence signal of a fluorophore captured in the hotspot of plasmonic nanoparticles (NPs). Resolving the latter two challenges requires maximizing the probability of capturing the target molecules in the shortest possible time. We address this with an integrated NACHOS‐diagnostic approach involving several steps of development (**Figure** **1**). These include optimization of the DNA origami NanoAntenna design; integrating nanopatterning and microfluidics; and engineering of a fluorescence reader with single‐molecule detection software.

### 2.1 NanoAntenna Design and Nucleic Acid Assay Optimization

**Figure 2:**
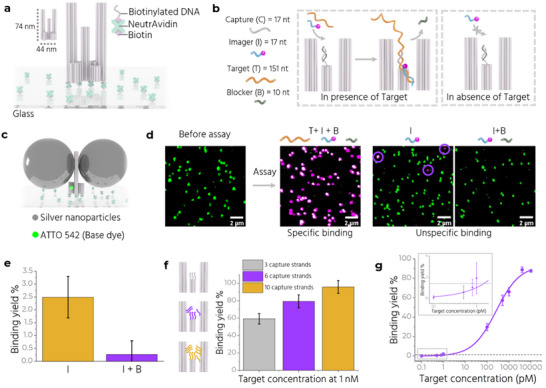
Optimization of nucleic acid assay detection with NACHOS. a) Sketch of the Trident nanostructure immobilized on a BSA‐Biotin‐NeutrAvidin modified glass coverslip. b) Working principle of the assay in presence and absence of the target. A single capture strand is shown for simplified visualization. c) Sketch of a fully assembled Trident NACHOS with 80 nm silver nanoparticles and an ATTO 542 dye at the base. d) Confocal fluorescence scans before and after the assay. Unspecific binding of the imager in absence of target is marked with purple circles. Scale bars: 2 µm. e) Binding yield or colocalization percentage comparison after incubation with imager only (in yellow) and with imager and blocker (in purple). f) (Left, insets) Schematic showing the positions of 3, 6 and 10 capture strands on the Trident. (Right) Target binding yield in the three cases upon incubation with target, imager and blocker. g) Target binding yield at varying target concentrations. Inset shows a magnified view between 0.1 and 1 pM target concentration. For each data point, at‐least 3 (20 µm × 20 µm) confocal scans with at‐least 300 molecules per scan are analyzed. The error bars represent the standard deviation. The purple line represents logistic fit. The gray dashed line indicates the minimum detectable target concentration above the blank.

We use the second generation of NACHOS^[^ ^31^ ^]^ for the NanoAntenna assembly as its larger gap (19 nm) between side pillars compared to the first generation (6.5 nm)^[^ ^13^ ^]^, allows more room for integrating multiple capture strands (Figure 1, panel 1). The DNA origami structure, called *Trident*, consists of a central pillar (51 nm in height) flanked on two sides by longer pillars (74 nm in height). The pillars emerge from a 44 nm wide cross‐shaped base (**Figure** **2**a; Figure S1, Supporting Information). We modify the base of the Trident to extend twelve biotinylated single‐stranded DNA (ssDNA) extensions from the bottom face, which are used to immobilize the structure on glass coverslips using Biotin‐NeutrAvidin linkages (Figure 2a). The sandwich‐type hybridization assay^[^ ^13^ ^]^ involves four strands – capture, target, imager, and blocker (Figure 2b). The “capture” strands are extended staple strands that protrude from the Trident and hybridize to the “target” strand, which is 151 nucleotides (nt) long, via a 17 nt complementary sequence. The “imager” strand, labelled with Alexa Fluor 647, has a 17 nt sequence complementary to another part of the target. The “blocker” strand is shorter (10 nt) and is complementary to a portion of the capture strand. It prevents unspecific interaction between the imager and capture strands in the absence of the target (Figure 1, panel 2 and Figure 2b; Figure S2, Supporting Information;). The target, when added, can displace the blocker (Figure 2b). We utilize a synthetic target sequence specific to the OXA‐48 gene, used for diagnosing carbapenem‐resistant *Klebsiella pneumoniae* infection (Note S1.1, Supporting Information).^[^ ^32^ ^]^ The gram‐negative bacterium can cause various infections, including pneumonia and other of the bloodstream and urinary tract.^[^ ^33^ ^]^

We use 80 nm silver nanoparticles (AgNPs) functionalized with thiolated T_20_ strands for plasmonic enhancement. We modify each side pillar to incorporate six A_20_ extensions to capture two AgNPs, one on each pillar, assembling the NanoAntenna (Figure 2c). An ATTO 542 fluorophore at the base of the Trident acts as an internal reference to determine the position of the nanostructure during confocal measurements (Figure 2c). Detailed characterization of the NanoAntenna is included in the supporting information (Figure S3, Supporting Information). We assess the specificity and efficiency of the assay by taking single‐molecule confocal fluorescence scans before and after performing the assay. Initially, we observe only green spots corresponding to the reference ATTO 542 (Figure 2d). After incubation with target (4 nM), imager (12 nM), and blocker (12 nM) strands for 1 h at 37 °C and subsequent washing, we detect magenta (absence of reference dye due to limited labelling efficiency) and white spots (colocalization of two dyes on the structure) (Figure 2d). To quantify the assay, we calculate the specific ‘binding yield' as

(1) $$\mathrm{Binding}\;\mathrm{yield}\;=\frac{\mathrm{Number}\;\mathrm{of}\;\mathrm{magenta}\;\mathrm{and}\;\mathrm{white}\;\left(\mathrm{colocalized}\right)\;\mathrm{spots}}{\mathrm{Number}\;\mathrm{of}\;\mathrm{total}\;\mathrm{spots}}$$

To quantify ‘unspecific binding’, we perform the assay without the target. We obtain fewer colocalized spots when both imager and blocker are present compared to imager alone (Figure 2d,e).

As predicted, the larger gap between the two side pillars in the Trident allows integration of more capture strands in the hotspot region. Increasing the number of capture strands increases the binding yield (Figure 2f). Bleaching step analysis confirms that a single Trident NACHOS with 10 capture strands can capture up to 8 target strands at higher concentrations (4 nm) (Figure S4, Supporting Information). This supports the increased accessibility of the hotspot in the current design compared to the previous design, where more than 70% structures captured only a single target strand.^[^ ^13^ ^]^ We also examine whether including more capture strands affects NP binding due to steric hindrance. We compare Trident variants with zero and 10 capture strands, and do not observe a loss in enhancement with the introduction of more capture strands (Figure S5, Supporting Information). Hence, we employ 10 capture strands for subsequent experiments. Next, we measure the target binding yield at varying target concentrations from 100 fM to 10 nM (Figure 2g). The imager and blocker concentrations were kept constant at 12 nM for each sample (See Figure S6 for example fluorescence scans, Supporting Information). In this setting, the limit of detection (LoD) for the assay is determined to be ≈1 pM using the formula:

(2) LoD=Meanvalueofblank+3×StandardDeviationofblank

### 2.2 Optimizing the Cross‐Section for Binding with DNA Origami NanoAntenna Placement

**Figure 3:**
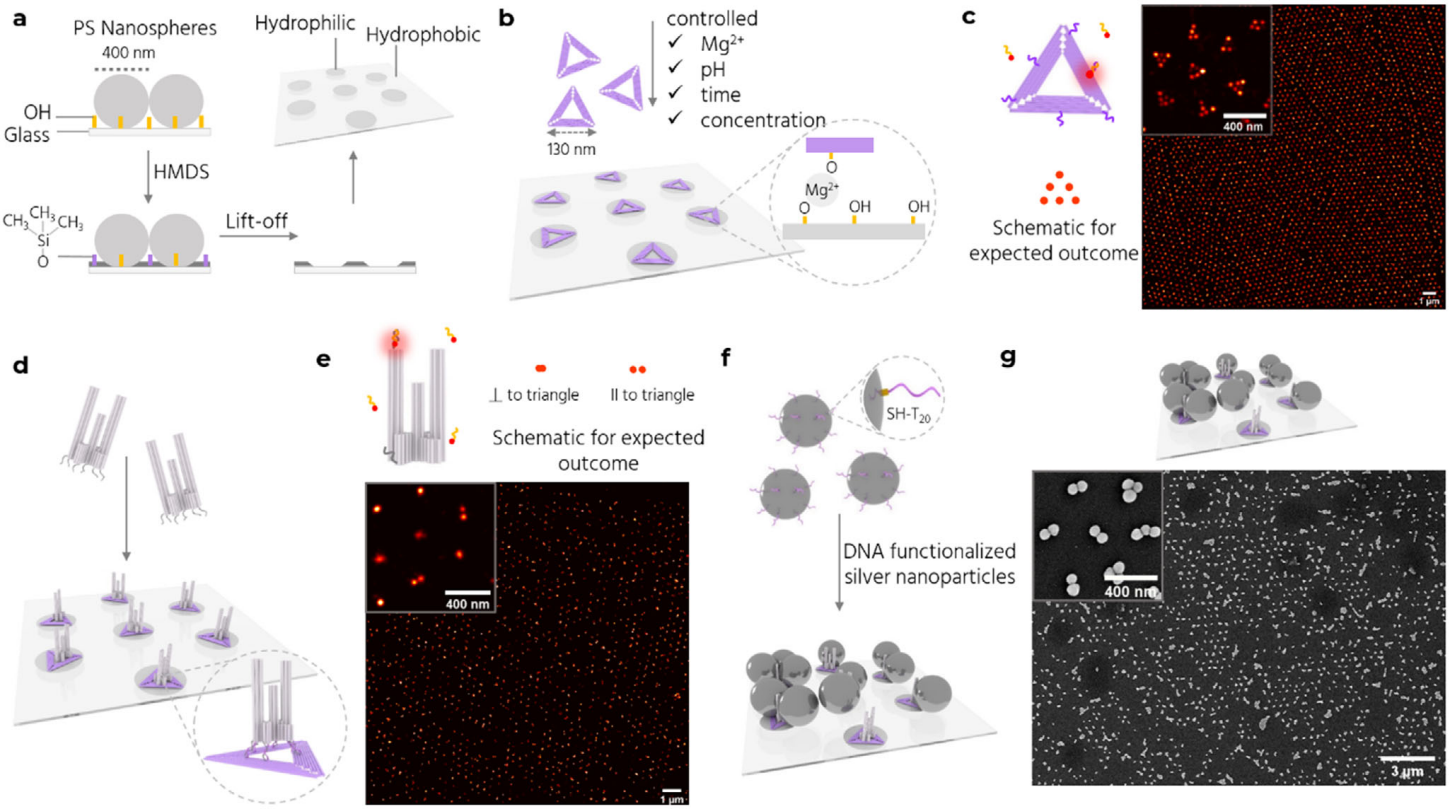
Site‐specific nanopatterning of Trident NanoAntennas. a) Fabrication of a nanopatterned surface via nanosphere lithography involving polystyrene (PS) nanospheres deposition, vapor‐phase passivation by hexamethyldisilazane (HMDS), and lift‐off. b) DOP of Triangle DNA origami nanostructures on the hydrophilic placement sites through electrostatic interactions between the placement sites and the Triangle (inset). c) (Left) Triangle with 6 docking sites (purple) for DNA‐PAINT experiments and a schematic of the expected outcome. (Right) DNA‐PAINT image of the triangle placed on a nanopatterned surface with a zoom‐in (inset). d) DNA hybridization between the Trident and the Triangle. e) Trident with 2 docking sites (dark gray) for DNA‐PAINT experiments and a schematic of the expected outcome (top) for upright Tridents (correct orientation) or fallen Tridents (undesired orientation) on the Triangle. A single strand per docking site is shown for simplicity. DNA‐PAINT image of the Trident bound to the Triangle is shown with a zoom‐in (inset). f) Incubation with functionalized AgNPs (dark gray spheres) results in full NanoAntenna assembly. g) SEM image of NanoAntennas on a patterned surface with a zoom‐in (inset).

Besides increasing the cross‐section for target binding by increasing the number of capture strands per NanoAntenna, we aim next at increasing the surface density of NanoAntennas. The challenge here is to achieve dense placement of NanoAntennas on a substrate without them interacting with each other and aggregating into clusters with uncontrolled properties.^[^ ^34^ ^]^ We adapt the combination of DNA origami placement (DOP)^[^ ^35^, ^36^, ^37^ ^]^ and nanosphere lithography^[^ ^38^ ^]^ to regularly arrange NanoAntennas at 400 nm distances (Figure 1, panel 3). The method involves drop‐casting polystyrene nanospheres (400 nm diameter) onto a hydrophilic coverslip and then passivating the surface to render the unmasked areas hydrophobic. The spheres are then lifted off, revealing a hexagonal array of hydrophilic *placement sites* (**Figure** **3**a). DNA origami nanostructures are selectively placed on these sites through electrostatic interactions. A high‐quality DOP, defined as one origami per placement site, is achieved by optimizing Mg^2+^ concentration, pH, incubation time, and DNA origami concentration (Figure 3b). Our modified DOP protocol involves two placement steps. First, a 2D *Triangle* origami is placed on the substrate. We use a modified version of the “Rothemund triangle” with a side length of ≈127 nm (Figure S7, Supporting Information),^[^ ^39^ ^]^ which corresponds well to the placement sites of ≈120 nm diameter.^[^ ^35^ ^]^ Second, the Trident is introduced with six ssDNA strands extending from its bottom face that are complementary to the six protruding sequences on the Triangle. The two‐step placement prevents direct interaction of the Trident with placement sites, which otherwise results in the placement of multiple Tridents per placement site in random orientations (Figure S8, Supporting Information). We use atomic force microscopy (AFM) (Figure S9, Supporting Information) and DNA‐PAINT (Point Accumulation In Nanoscale Topography)^[^ ^40^, ^41^ ^]^ to characterize the quality of placement. DNA‐PAINT, as a super‐resolution microscopy technique, allows single‐molecule localization of freely diffusing short, labeled DNA probes (imager) transiently binding to complementary ssDNA (docking strand) on the origami. We incorporate six docking strands (60 nm apart) (Figure S10, Supporting Information) on the outer rim of the Triangle and perform DNA‐PAINT imaging with an 8 nt imager labelled with ATTO 655. We observe a hexagonal placement pattern with Triangles placed ≈400 nm apart (Figure 3c). We also observe geometric defects consistent with those observed in scanning electron microscopy (SEM) images of self‐assembled nanospheres on glass (Figure S11, Supporting Information).

After the Triangles are placed, we add the Trident. The extensions at the base of the Trident hybridize with the protrusions on the Triangle (Figure 3d). The extensions are placed at the inner hole, protruding in the plane of the Triangle, to make sure they are accessible irrespective of which face of the Triangle lands on the placement site. We use DNA‐PAINT to study the orientation of the Tridents after placement by incorporating six docking strands (three near the top and three near the bottom of one outer pillar). Tridents in the desired upright orientation are observed as one overlapping spot, while those lying parallel to the surface show up as two spots (Figure 3e). We achieve more than 80% upright Tridents after optimizations (Figure S12, Supporting Information).

Next, we incubate the patterned Tridents with thiolated‐DNA functionalized 80 nm AgNPs to complete the NanoAntenna assembly (Figure 3f). Under ideal conditions, each NanoAntenna would contain two AgNPs to create a “dimer.” To achieve this, it is imperative to refine the functionalization protocol to minimize NP aggregation (Figure S13, Supporting Information). Although aggregation does not compromise the assay and can even yield elevated enhancement factors,^[^ ^42^ ^]^ it poses a challenge for achieving uniform, closely spaced assemblies. We employ the freeze and thaw functionalization method to increase the DNA loading on each NP,^[^ ^43^ ^]^ followed by an agarose gel‐based purification step to separate single AgNPs from aggregates and free thiol‐DNA strands. SEM imaging reveals the hexagonal array of NanoAntennas on the coverslips (Figure 3 g; Figure S14, Supporting Information). As a control, we perform DOP with Triangles and incubate the surface with functionalized NPs, observing minimal or no binding. This confirms the selective binding of NPs to the Trident (Figure S15, Supporting Information). Adding Tridents directly to a surface with empty placement sites results in the placement of multiple Tridents per placement site, many of which are misoriented or have fallen. Adding AgNPs to this sample results in minimal binding or aggregation (Figure S16, Supporting Information). This suggests that it is crucial to have at least one Trident bound to the Triangle in the desired orientation for proper assembly of the NanoAntenna. Additional Tridents bound to the Triangle in random orientations are unable to capture NPs to form the plasmonic hotspot due to steric hindrance and are rendered unfunctional.

### 2.3 Single‐Molecule Fluorescence Reader

**Figure 4:**
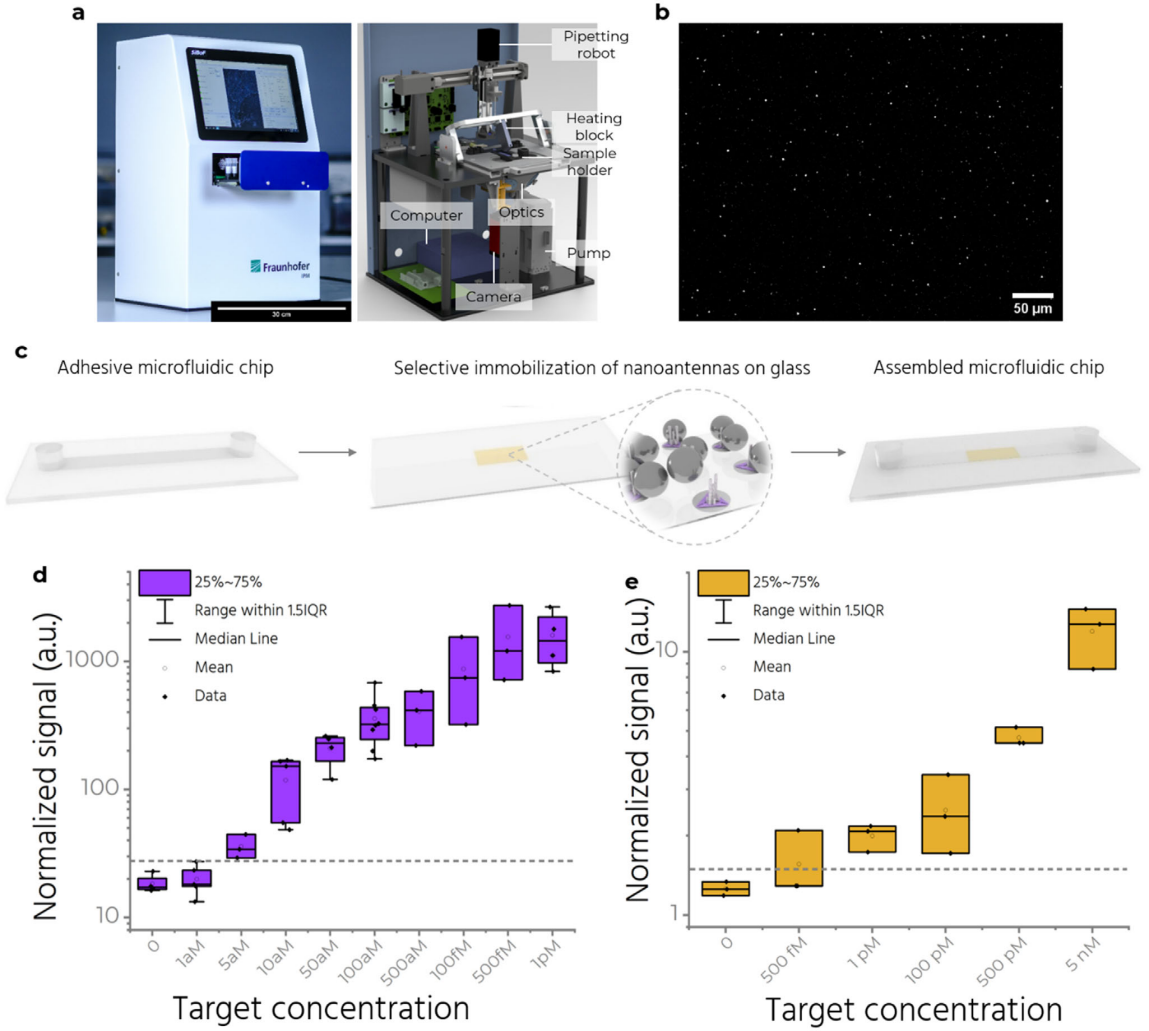
Microfluidics and a simple fluorescence reader. a) (left) The single‐molecule fluorescence reader and (right) a sketch showing its components. Scale bar: 30 cm. b) A zoom‐in view of an exemplary image captured on the reader after sandwich assay with target concentration of 1 pM. Scale bar: 50 µm. c) A self‐adhesive microfluidic chip is attached on top of the coverslip with patterned NanoAntennas. The yellow rectangle depicts the area where DOP of NanoAntennas is performed. d) Measurements showing efficiency of the assay at target concentrations from 1 aM to 1 pM, performed on the reader and analyzed by spot picking. The gray dashed line indicates the minimum target concentration detectable above the blank. e) Measurements on the reader analyzed by intensity averaging between target concentrations of 500 fM to 5 nm. The box plots in (d) and (e) show the 25/75 percentiles, and the whiskers represent the 1.5×IQR (inter quartile range) length, the center lines represent the average values. The gray dashed line indicates the minimum target concentration detectable above the blank in (d) and (e).

To enable POC detection of single molecules on a cost‐efficient device that also provides a large field‐of‐view (FOV) to count all the single molecules captured by NanoAntennas, we developed a fluorescence reader (**Figure** **4**a and Figure 1, panel 4). The reader uses four spectrally filtered LEDs to excite the Alexa Fluor 647 and a CMOS camera for detection. It is sensitive enough to detect single molecules when their fluorescence is enhanced by NanoAntennas (Figure 4b; Figure S17, Supporting Information). At our standard settings of 2 W cm^−2^ excitation power density and an integration time of 300 ms, single molecules not enhanced by NanoAntennas remain dark. This creates an intrinsic filter against false positive signals, as unspecific binding in the ultra‐small volumes of the NanoAntenna hotspot is minimal, whereas an unavoidable background of not‐enhanced single‐molecule impurities is invisible.^[^ ^13^ ^]^ The reader also includes two white LEDs to collect scattering light from NPs. An integrated computer with a touchscreen serves as the user interface, running custom software that can control all components and execute script‐based processes. The software also performs image processing to detect single molecules based on their intensity and temporal blinking behavior. A detailed description of the reader specifications, data acquisition and analysis, as well as the costs involved, is provided in Note S2 (Supporting Information).

For finding all target molecules in our 100 µL sample solutions, the reader features an FOV of 3 mm × 2.5 mm. This matches the self‐adhesive microfluidic chip that features a channel (Width: 2.5 mm, Depth: 150 µm, Length: 58.5 mm), inlet and outlet ports for easy pipetting, and can attach to glass substrates (Figure 4c). This selective placement (Figure S18, Supporting Information) allows us to detect most of what we capture, minimizing the loss of target strands outside the detection FOV. Besides the high density of capturing strands and the increased density of NanoAntennas, the large FOV further enhances the interactions of the sample volume with the NanoAntennas. Next, we employ fluidics housed in the reader‐ a simple, automated repetitive back‐and‐forth flow within the narrow channel in the microfluidic chip, to further increase the binding kinetics beyond purely diffusive interactions (Figure 1, panel 5). Applying flow allows us to achieve a ≈10‐fold increase in target capture. We demonstrate this by comparing assays on chips patterned with NanoAntennas and comparing the two cases‐ one performed with flow and the other without (Figure S19, Supporting Information), both with an incubation time of 1 h and a target concentration of 500 fM.

### 2.4 Achieving Attomolar Sensitivity

Combining the various elements (Figure 1, panels 1–5) into the NACHOS chip, we use the reader to characterize assays performed at target concentrations ranging from 1 aM to 5 nM, achieving an LoD of ≈5 aM (Figure 4d,e; Figure S20, Supporting Information).

We determine this by counting the number of spots detected in each sample, normalized to the Nanoantenna surface density (see S2.3, Supporting Information). Measurements were repeated at least three times to obtain a standard deviation. In our reader, with an optical resolution of ≈2.7 µm (S2.1, Supporting Information), a single molecule is detected as a spot (Point Spread Function) with a diameter of 3 pixels, with each pixel corresponding to 0.8 µm. Within the aM‐fM concentration range, the probability of spatially overlapping signals from two different target molecules remains low, so each detected spot is assumed to correspond to a single molecule. Specifically, at 1 pM, we estimate the average proportion of double molecules among the detected spots to be 0.5–1.5% (S2.5, Supporting Information). Thus, throughout the aM–fM range, the proportion of unaccounted double molecules is safely below 1%. At concentrations above 1 pM, the spots are too closely spaced to accurately distinguish them individually (Figure S34, Supporting Information). We therefore use an intensity‐based analysis, which calculates an average intensity value over the whole image – to analyze data from 500 fM to 5 nm. Using average intensity alone, we calculate a LoD of ≈1 pM, further highlighting the advantage of single‐molecule counting over averaging. It is worth noting that the combination of the two detection strategies (in their respective ranges of applicability) preserves accuracy in the full concentration range, from aM to nM regime.^[^ ^44^ ^]^

### 2.5 Silicification for Stability in Clinically Relevant Fluids

**Figure 5:**
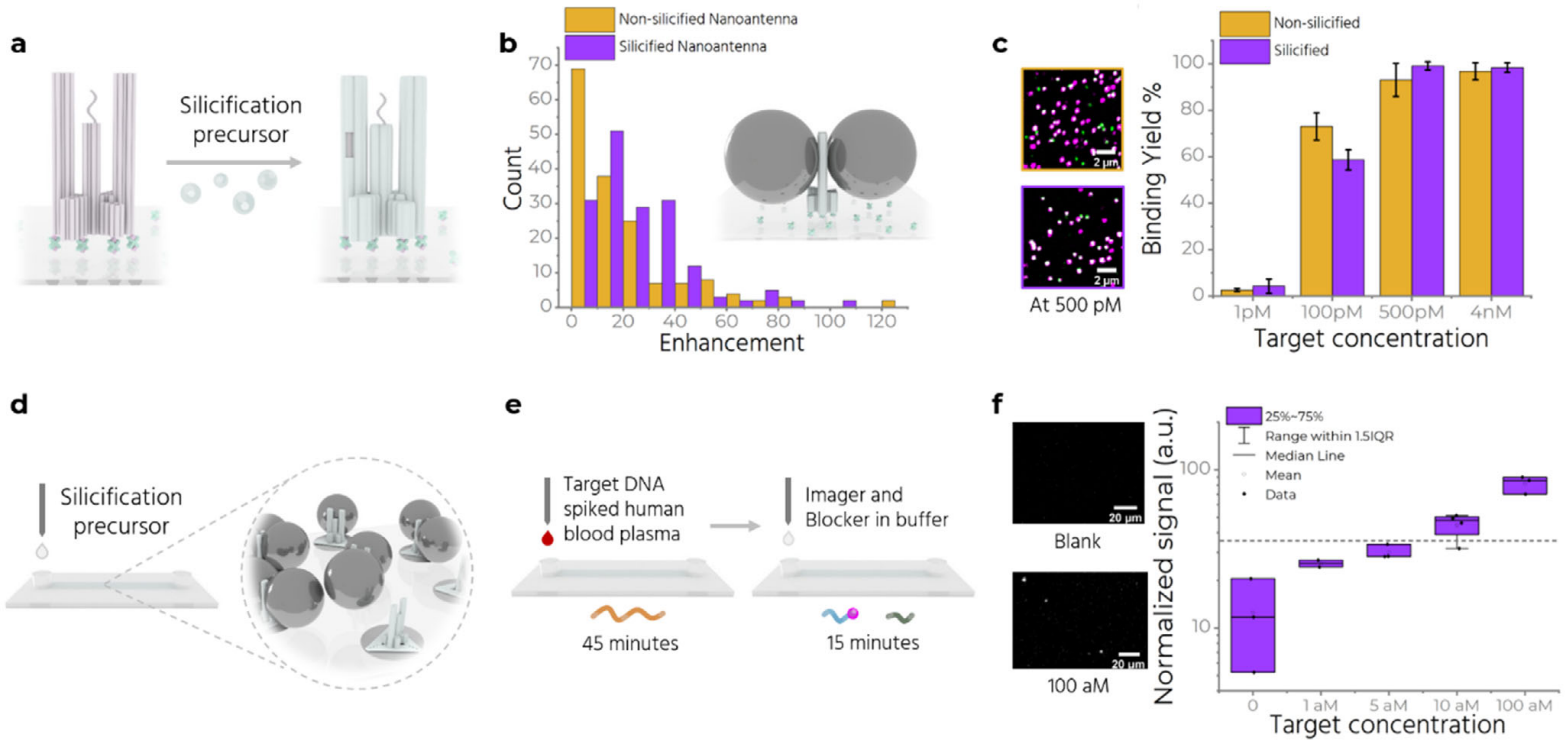
Silicification and measuring in human blood plasma. a) Coating of the double‐stranded regions of the Trident with silica. b) Enhancement values are compared for non‐silicified and silicified NanoAntennas. (inset) Schematic of a silicified NanoAntenna. c) (left) Exemplary confocal fluorescence scans for silicified and non‐silicified NanoAntenna after incubating with 500 pM target concentration on the left. (right) Column plot comparing target binding yield at different target concentration in case of silicified and non‐silicified NanoAntennas. d) Silicification of nanopatterned NanoAntennas assembled in the microfluidic chip. e) The microfluidic chip is incubated first with target spiked plasma for 45 min, washed with buffer, and then incubated with imager and blocker for 15 min. f) Measurements on the reader with target spiked plasma at target concentrations 0, 1, 5, 10 and 100 aM, with silicified NanoAntennas. Gray dashed line indicates the lowest detectable signal above blank. Zoom‐in snapshots from the blank and 100 aM chip is shown on the left. Scale bar at 20 µm.

Biological fluids like plasma contain many components in addition to target molecules. These components can interact unspecifically with substrates and add to the noise, reducing the sensitivity of our assay. We use buffers containing monovalent electrostatic ions like Na^+^ to reduce such interactions and wash away weakly‐bound unspecific molecules (Figure S21, Supporting Information). But the same Na^+^‐containing buffers also lift off the DNA origami structures placed through DOP (Figure S22, Supporting Information). To overcome these issues, we employ silicification (Figure 1, panel 6), which involves the formation of a robust silica coating on the DNA origami nanostructure. The inorganic coating significantly enhances the stability of the structure and keeps them “glued” to the surface (Figure S23, Supporting Information) over a wide range of pH, temperature, and salt concentrations.^[^ ^45^, ^46^ ^]^ Importantly, silicification does not affect the accessibility of ssDNA extensions (capture strands), which is crucial for the assay's functionality (**Figure** **5**a).^[^ ^47^ ^]^

We introduce a pre‐hydrolyzed precursor – prepared by mixing *N*‐trimethoxylsilylpropyl‐*N*,*N*,*N*‐trimethylammonium chloride (TMAPS) and tetraethoxysilane (TEOS) solutions in Mg^2+^ buffer^[^ ^48^ ^]^ to the surface with DNA origami, incubate for desired durations, wash with water and ethanol, and air‐dry to complete the process. We use AFM to compare the height of the base of the Trident prior to silicification, and after one and four days of silicification to determine optimal silicification parameters (Figure S24, Supporting Information). We observe a thickness increase of ≈3 nm after one and ≈7 nm after four days of silicification, consistent with other studies.^[^ ^47^, ^48^ ^]^ After 4 days of silicification, we could not reasonably resolve the structural features of the Trident with AFM, and observed an increase in the formation of unidentified clusters. We chose the 1‐day incubation period for further experiments.

Next, we check the effect of silicification on fluorescence enhancement by silicifying NanoAntennas immobilized on glass using BSA‐biotin‐NeutrAvidin. These NanoAntennas are folded with an Alexa Fluor 647‐modified staple strand, which positions the dye in the hotspot, in addition to the base dye (ATTO 542). We record single‐molecule confocal fluorescence scans and measure the intensity of the Alexa Fluor 647, computing a maximum enhancement factor of 126 and 102 for non‐silicified and silicified NanoAntennas, respectively (Figure 5b). We also check if silicifying the origami adversely affects the accessibility of the capture strand in the hotspot. Results show similar target‐capture binding yields in silicified and non‐silicified NanoAntennas at all tested target strand concentrations (Figure 5c). We then test the performance of silicified NanoAntennas in untreated human blood plasma. Plasma – a clear, slightly yellow liquid composed of blood without red blood cells, white blood cells, and platelets is ubiquitously used in diagnostics.

We start by silicifying NanoAntennas immobilized using BSA‐biotin‐NeutrAvidin chemistry. We then incubate the sample for 1 h with plasma spiked with 5 nM of target and 12 nM of both imager and blocker and measure the imager and base dye colocalization (Figure S25, Supporting Information). We observe only green spots, consistent with the base dye, indicating the presence of stable origami on the surface. Absence of other colored spots suggests degradation of the target and/or imager by enzymes in the plasma. Next, we perform an assay under the same buffer conditions by incubating with plasma spiked only with the target for an hour, washing the sample with buffer to remove the plasma, and then incubating with the imager and blocker for 15 min (Figure S25, Supporting Information). We observe colocalized spots, confirming proper functioning of the assay and suggesting that the imager gets quickly degraded in plasma but not the target. Additionally, we introduce 1 µM of random DNA sequence (sacrificial DNA) during the target incubation. This results in a 94.6% binding yield, an improvement over the 90.8% yield without sacrificial DNA (Figure S25, Supporting Information). The target's longer sequence (151 nt) and its tendency to form secondary structures^[^ ^49^ ^]^ make it less prone to enzymatic degradation (Figure S26, Supporting Information). Shorter sacrificial DNA strands appear to further slow this degradation process. We adapt this strategy and incubate the NACHOS chip (Figure 5d) with target‐spiked plasma for 45 min, put it in the reader with fluidics, and wash the chip to remove any unspecifically bound residues originating from the plasma. We then re‐incubate the chip with the imager and blocker for 15 min while performing fluidics, and wash with fresh buffer (Figure 5e). We measure target concentrations from 1–100 aM and observe the lowest detectable concentration above the blank to be ≈10 aM after silicification (Figure 5f; S27, Supporting Information), compared to 5 aM in assays performed with unsilicified NanoAntennas. Silicification thus extends the stability of NanoAntennas to clinically relevant fluids without impacting sensing performance (see Figure S28 for discussion on NanoAntenna stability and storage, Supporting Information).

Finally, we use toehold‐mediated strand‐displacement^[^ ^50^ ^]^ to displace the bound target‐imager duplex from the capture strand, making the capture strand accessible for the next round of use (Figure 1, panel 7; Figure S29, Supporting Information). As the cost of a diagnostic test often determines its accessibility to those most in need, making the chips reusable and offering users the ability to modify the same chip for detecting various target sequences can benefit POC applications.

## 3 Conclusion

In this work, we have developed an amplification‐free POC‐compatible nucleic acid detection assay capable of attomolar sensitivity, targeting a 151 nt sequence specific to antibiotics‐resistant *Klebsiella pneumoniae*. DNA origami NanoAntennas, particularly NACHOS, excel in distinguishing signals from background noise. We merged the strengths of NACHOS with nanopatterning and microfluidics to offer an integrated approach that addressed key challenges in detecting low‐abundance targets.

A nanopatterned surface densely populated with NanoAntennas combined with microfluidics to overcome the effects of slow kinetics at low target concentrations, increases the probability of target capture. We develop a single‐molecule fluorescence reader boasting a larger FOV to efficiently detect captured targets. Silicification reduces the dependence of our system on ionic concentrations, pH, temperature as well as protects against degrading enzymes. Finally, a strand displacement strategy is optimized to be able to re‐use our chips. We use a sandwich‐type assay to detect target DNA bearing the sequence specific to a clinically relevant microbe and achieve a detection limit of 5 am in buffer and 10 aM in untreated human blood plasma within 1 h. It should be possible to modify our chip to target many different DNA or RNA targets, by extending an “adapter” sequence from the origami to which a part of a capture strand can bind, allowing the user to choose the sequence for the capture and thus the target.

Established molecular diagnostic methods such as Reverse Transcription Polymerase Chain Reaction (RT‐PCR) typically achieve LoDs ≈10–100 aM but require enzymatic reverse transcription, thermal cycling, and assay times of 60–120 min.^[^ ^51^ ^]^ Loop‐mediated isothermal amplification (LAMP), reports LoDs in the range of ≈10 fM–100 aM with faster assay times (≈30–60 min), but it is prone to false positives and offers limited multiplexing due to primer design constraints.^[^ ^52^ ^]^ Clustered Regularly Interspaced Short Palindromic Repeats or CRISPR‐based methods provide high multiplexing potential but must combine enzymatic amplification (e.g., Recombinase Polymerase Amplification (RPA) or RT‐RPA) with Cas‐based detection to reach LoDs as low as ≈1–10 aM.^[^ ^28^ ^]^ In contrast, NACHOS achieves comparable or superior sensitivity (≈5 aM) without enzymatic amplification or pre‐concentration steps. The total assay time is ≈45–60 min with most potential for acceleration by improved turbulent mixing. While the current assay time is similar to established methods, the enzyme‐free nature and multiplexing potential (via multi‐color imaging and multi‐site addressable DNA origami) offer distinct advantages for practical point‐of‐care use.

Overall, our method offers a robust and scalable solution for sensitive nucleic acid detection in various clinical settings, with potential applications in diagnosing antibiotic‐resistant infections, cancer biomarkers, and neurodegenerative diseases. By providing a simple, amplification‐free approach with high sensitivity and specificity, our platform addresses critical needs in rapid, on‐site diagnostics, potentially improving patient outcomes and aiding in the global fight against antimicrobial resistance. Finally, NACHOS‐diagnostics has the potential to evolve into an alternative to digital PCR for quantifying nucleic acid molecules, as it may eliminate the need for separation into small reaction vessels.

## 4 Experimental Section

### DNA Origami

DNA origami structures were designed using caDNAno2,^[^ ^53^ ^]^ and assembled and purified following protocols adapted from Wagenbauer et al.^[^ ^54^ ^]^ A mixture containing 30 nM of in‐house produced p8064 scaffold for Trident and p7249 for Triangle, 300 nM unmodified staples (pooled from an original 100 µM concentration), and 750 nM modified staples (also pooled from 100 µM) was prepared. Staples were supplied by Integrated DNA Technologies Europe GmbH, Germany; Eurofins Genomics GmbH, Germany, and biomers.net GmbH, Germany, and their exact sequences are listed in Note S3 (Supporting Information). 10 × folding buffer (FoB) (200 mM MgCl_2_, 50 mM Tris, 50 mM NaCl, 10 mM EDTA) was added, and thermal annealing ramp was performed over 16.5 h (detailed in Table S5, Supporting Information). Purification was conducted using 100 kDa MWCO Amicon Ultra filters (Merck KGaA, Germany), with four washes in low ionic strength buffer (5 mm MgCl_2_, 5 mM Tris, 5 mM NaCl, 1 mM EDTA) for 5 min at 10 000 × g, 4 °C. The filter was inverted and the purified origami collected in a fresh tube after centrifuging at 1000 × g for 5 min.

### Nanoparticle Functionalization

A freeze and thaw approach was adapted from Liu et. al.^[^ ^43^ ^]^ Typically, 100 µL of 1 mg ml^−1^ 80 nm BioPure AgNPs (nanoComposix, USA) were transferred into a low‐bind 1.5 mL Eppendorf tube. Two tubes of lyophilized thiol‐modified ssDNA (5′‐T_20_‐SH‐3′, 4 nmol, Ella Biotech GmbH) were reconstituted in 670 µL of nuclease‐free water each. The thiolated‐DNA strands were gradually added to the NPs while mixing with a pipette. Next, 60 µL of 5 m NaCl was introduced and mixed gently. The mixture was then frozen at −20 °C for a minimum of 1 h. For purification, the mixture was thawed and centrifuged at 2800 × g, 4 °C for 15 min. After discarding the supernatant, 1× BlueJuice loading dye (Merck, Germany) was added and mixed by pipetting. The sample was loaded on a 1.2% agarose gel and electrophoresis was run at 100 V for 45 min in an ice bath, to isolate the monomer functionalized NPs from aggregates and excess ssDNA. The monomer band was excised, squeezed, mixed with an equal volume of nuclease‐free water, and centrifuged under the same conditions. The supernatant was removed, the concentration measured at Nanodrop 2000 (Thermo Fisher, USA), and the sample stored at 4 °C for later use.

### Surface Preparation for Immobilizing NanoAntennas with Biotinylated Strands

Microscope coverslips measuring 24 mm × 60 mm and 170 µm thick (Carl Roth GmbH, Germany) were rinsed with Milli‐Q water and isopropanol, dried using an air stream, and subsequently treated in a UV‐Ozone cleaner (PSD‐UV4, Novascan Technologies, USA) for 30 min at 100 °C. SecureSeal Hybridization Chambers (2.6 mm depth, Grace BioLabs, USA) were applied to the cleaned coverslips. The chambers were washed thrice with Phosphate‐buffered saline (PBS) and incubated for at least 5 min with BSA‐biotin (0.5 mg mL^−1^, Sigma Aldrich, USA), followed by 5 min with NeutrAvidin (0.2 mg mL^−1^, Thermo Fisher Scientific, USA). Trident DNA origami (100 pM) (in 5 mM Tris, 2 m NaCl, 1 mM EDTA) with twelve biotinylated ssDNA extensions was added to bind NeutrAvidin; excess origami was removed after 1.5 min. Next, functionalized NPs were added at 0.05 OD (measured at a path length of 1 mm) and incubated overnight in the dark at room temperature in the buffer (5 mM Tris, 2 m NaCl, 1 mM EDTA). The following day, samples were washed 4–5 times with the same buffer to remove unbound NPs.

### Nucleic Acid Assay

Trident DNA origami with 10 capture strands protruding from the hotspot was folded to detect a 151 nt synthetic DNA sequence specific to the OXA‐48 gene carrying an antibiotic resistance (see Note S1.1, Supporting Information). The NanoAntennas were assembled as mentioned above and incubated with 4 nM target strand, 12 nM Alexa Fluor 647‐ labeled imager strand (17 nt), and 12 nM blocker strand (10 nt) in a buffer containing 5 mM Tris, 2 m NaCl, 1 mM EDTA, at 37 °C for 1 h. After incubation, the unbound strands were washed off with the same buffer.

### Surface Preparation for Patterned NanoAntennas

A modified DNA origami placement method was adapted from Shetty et. al.^[^ ^35^ ^]^ Microscope coverslips of 25 mm × 75 mm and 170 µm thick (Electron Microscopy Sciences, Pennsylvania, USA) were rinsed with Milli‐Q water and isopropanol, then dried with an air stream. A rectangle of ≈7 mm × 5 mm was marked with a marker in the center of one side and the reverse side was used for sample preparation. The unmarked area was covered with extra glass coverslips, and the marked region was treated with UV‐Ozone cleaner for 30 min at 100 °C. A 350 µL suspension of 400 nm polystyrene nanospheres (Thermo Scientific Nanosphere Size standard 3400A) was centrifuged for 5 min at 10 000 × g at room temperature, the supernatant discarded, and the pellet re‐suspended in 350 µL of Milli‐Q water. This wash was repeated twice. The final pellet was re‐suspended in 100 µL of 25% ethanol/water and 10 µL was drop‐cast onto the marked area while keeping the coverslip at a ≈30° tilt. After heating at 60 °C for 4–5 min, the coverslips were exposed to HMDS vapors for 30 min in a sealed glass chamber. Spheres were lifted‐off by ultrasonication in water, dried under nitrogen, and annealed at 120 °C for 5 min. The Triangle DNA origami was diluted to 150 pm and ≈200 µL was added to the marked area, and incubated for 1 h at room temperature in placement buffer (PB, 40 mM Tris, 40 mM MgCl_2_ at pH≈8.4). After washing with PB and 0.05% Tween20, 350 pM Trident DNA origami was added and incubated for another 1 h, followed by repeated washing. Finally, 50 µL of functionalized 80 nm AgNPs (0.2 OD, measured at 1mm path length) in PB with Tween20 were added and incubated overnight in the dark. The coverslips were washed 5–6 times in PB containing Tween20.

### Sample Preparation for Measurements On The Reader

The patterned area was kept moist, and an adhesive microfluidic chip (Straight channel chip with adhesive tape, Fluidic 268, channel width: 2500 µm, channel depth: 150 µm, channel length: 58.5 mm, material: Zeonor, from microfluidic ChipShop GmbH, Germany) was mounted on the coverslip. The channel was flushed with PB containing Tween20 prior to measurements.

## Conflict of Interest

Philip Tinnefeld is an inventor on an awarded patent of the described bottom‐up method for fluorescence enhancement in molecular assays, EP1260316.1, 2012, US20130252825 A1. The remaining authors declare no competing interests.

## Author Contributions

The manuscript was written through the contributions of all authors. All authors have given approval to the final version of the manuscript.

## Supporting information



Supporting Information

## Data Availability

The data that support the findings of this study are available in the supplementary material of this article.
